# Comparative Proteomics of Activated THP-1 Cells Infected with *Mycobacterium tuberculosis* Identifies Putative Clearance Biomarkers for Tuberculosis Treatment

**DOI:** 10.1371/journal.pone.0134168

**Published:** 2015-07-27

**Authors:** Benjawan Kaewseekhao, Vivek Naranbhai, Sittiruk Roytrakul, Wises Namwat, Atchara Paemanee, Viraphong Lulitanond, Angkana Chaiprasert, Kiatichai Faksri

**Affiliations:** 1 Department of Microbiology and Research and Diagnostic Center for Emerging Infectious Diseases (RCEID), Faculty of Medicine, Khon Kaen University, Khon Kaen, Thailand; 2 Nuffield Department of Medicine, University of Oxford, Oxford, United Kingdom; 3 Centre for the AIDS Program of Research in South Africa, Nelson R Mandela School of Medicine, University of KwaZulu Natal, KwaZulu Natal, South Africa; 4 National Center for Genetic Engineering and Biotechnology (BIOTEC), National Science and Technology Development Agency (NSTDA), Rama VI Rd., Pathumthani, Thailand; 5 Department of Microbiology, Faculty of Medicine Siriraj Hospital, Mahidol University, Bangkok, Thailand; University of Palermo, ITALY

## Abstract

Biomarkers for determining clearance of *Mycobacterium tuberculosis (Mtb)* infection during anti-tuberculosis therapy or following exposure could facilitate enhanced monitoring and treatment. We screened for biomarkers indicating clearance of *Mtb* infection *in vitro*. A comparative proteomic analysis was performed using GeLC MSI/MS. Intracellular and secreted proteomes from activated THP-1 cells infected with the *Mtb* H37Rv strain (MOI = 1) and treated with isoniazid and rifampicin for 1 day (infection stage) and 5 days (clearance stage) were analyzed. Host proteins associated with early infection (n = 82), clearance (n = 121), sustained in both conditions (n = 34) and suppressed by infection (n = 46) were elucidated. Of the potential clearance markers, SSFA2 and CAECAM18 showed the highest and lowest protein intensities, respectively. A western blot of CAECAM18 validated the LC MS/MS result. For three clearance markers (SSFA2, PARP14 and PSME4), *in vivo* clinical validation was concordantly reported in previous patient cohorts. A network analysis revealed that clearance markers were enriched amongst four protein interaction networks centered on: (i) CD44/CCND1, (ii) IFN-β1/NF-κB, (iii) TP53/TGF-β and (iv) IFN-γ/CCL2. After infection, proteins associated with proliferation, and recruitment of immune cells appeared to be enriched possibly reflecting recruitment of defense mechanisms. Counteracting proteins (CASP3 vs. Akt and NF-κB vs. TP53) associated with apoptosis regulation and its networks were enriched among the early and sustained infection biomarkers, indicating host-pathogen competition. The BRCA1/2 network was suppressed during infection, suggesting that cell proliferation suppression is a feature of *Mtb* survival. Our study provides insights into the mechanisms of host-*Mtb* interaction by comparing the stages of infection clearance. The identified clearance biomarkers may be useful in monitoring tuberculosis treatment.

## Introduction

Tuberculosis (TB) is one of the greatest public health problems. One-third of the global population is infected with *Mycobacterium tuberculosis* (*Mtb*), the causative agent of TB. Despite tremendous efforts that have been directed toward controlling TB, TB still results in substantial morbidity and mortality.

Assessing infection clearance remains challenging yet is central to TB control. First, infection clearance is likely a frequent outcome following exposure because only 10–50% of close contacts with TB patients develop a positive tuberculin skin test (TST) and/or interferon gamma release assay (IGRA) after exposure [1]. The TST/IGRA non-responders may represent either persons who are truly unexposed to *Mtb* infection or those who cleared infection through an effective innate immune response or a localized immune response to infection without a positive TST and/or IGRA [2, 3]. Understanding the mechanism of primary clearance could assist in developing prophylactic interventions; however, identifying these individuals is challenging. Second, although TST/IGRAs are used to identify individuals with latent infection who are at a 2-3-fold elevated risk for reactivation [4], treating a latent TB infection does not result in TST/IGRA reversion [5]. Therefore, assessing latent TB treatment success is not currently possible. Third, the treatment of active TB usually consists of combined drug therapy for a minimum of 6 months for pulmonary disease or 9 months for extra-pulmonary disease. Microbiological clearance is typically determined by clinical and radiological improvement supplemented by sputum microscopy. However, microscopic analysis lacks sensitivity even in patients with active disease and is likely to be even less sensitive in a partially treated infection with low bacterial loads. The failure to achieve clearance may lead to relapse and to drug resistance evolution. Moreover, the assessment of newer, shorter-course regimens in development and testing [6, 7] would be aided by the availability of a reliable clearance marker.

Recent ‘omics’ studies have reported progress in identifying diagnostic biomarkers for active TB and predictive biomarkers of incident TB [8]. These biomarkers have largely been cellular or transcriptomic markers; however, protein biomarkers are among the first described [9]. Subsequent proteomic studies have predominantly focused on identifying markers to diagnose active disease [10–15]. In contrast, de Groote et al. [16] reported a targeted assessment of serum proteins in patients with active disease whose expression changed following 8 weeks of anti-TB therapy. However, the linkage to the microbiological response was not reported, the sterilization of infection was not ensured, and the proteins that are differentially expressed appear to be markers of reduced inflammation associated with clinical improvement, illustrating the complexities of biomarker discovery in complex organisms.

We screened for candidate biomarkers of *Mtb* clearance using an *in vitro* activated THP-1 cells model of treated TB infection and proteomic approaches.

## Materials and Methods

### THP-1 cell culture and activation

The human monocytic cell line THP-1 was cultured in RPMI 1640 supplemented with 2 mM L-glutamine and 10% FBS (Hyclone, GE Healthcare Life Science, UK). THP-1 cells (1.5 x 10^6^ cells/well) were activated using 50 nM/μl phorbol myristate acetate (PMA) and incubated at 37°C in a 5% CO_2_ atmosphere for 24 hr. To reduce the effect of PMA from THP-1 cells, the medium was replaced, and incubation was continued for another 24 hr.

### *Mtb* culture and inoculum preparation

*Mtb* H37Rv was grown in Middlebrook 7H9 with an OADC supplement for 14 days, adjusted to 0.5 McFarland standards with PBS and diluted with RPMI medium. *Mtb* cell suspension dispersion was achieved by repeated passage through a 26-gauge needle set.

### Infection experiments

After discarding the medium from THP-1 cells, *Mtb* H37Rv (1.5 x 10^6^ cells/well, MOI = 1) in cRPMI suspension was immediately refilled and then incubated at 37°C in a 5% CO_2_ atmosphere for 4 hr. After the infected cells were incubated, they were treated with 3 μg/ml isoniazid (INH) and 9 μg/ml rifampicin (RIF) (optimized drug concentration for killing *Mtb* within 3 days post-infection, which is consistent with microbiological clearance, S1 Table) in fresh RPMI medium. These concentrations approximate therapeutic drug concentrations that accumulate in patient serum [17]. Fresh drug-supplemented medium was fully exchanged every 24 hr until 5 days post-infection. The cell lysates and culture supernatants of the infected cells or uninfected cells treated with the same anti-TB drugs were collected after 1 day (infection stage) and 5 days (clearance stage) post-infection. We confirmed complete killing of *Mtb* in culture using aliquots of each specimen (both intra- and extra-cellular preparations). By 3 days (72 hr) post-infection, no *Mtb* growth was detected by colony forming unit (CFU) determination. Uninfected cells treated with anti-TB drugs were used as background controls. Three independent experiments were performed on different days as biological replicates.

### Proteome collection

Proteomes were collected from culture supernatants and cell lysates. For extracellular proteins, 3 ml of supernatant was collected from each well and filtered using a syringe filter (Whatman, GE Healthcare Life Science, UK). For the intracellular proteins, the infected cells were washed 2 times with phosphate-buffered saline (PBS) and scraped off using 500 μl PBS/well and transferred into conical tubes. Then, SDS (0.5% w/v) was added and incubated for 5 min, and the cells were sonicated at 53 KHz at 37°C for 30 min.

### Protein extraction and preparation

Two volumes of acetone were added to the culture supernatants and cell lysate suspensions. Protein suspensions were incubated at -20°C for 8 hr and then centrifuged at 8,000×*g* for 30 min at 4°C. Dried protein pellets were resuspended in 30 μl of sample storage buffer (50 mM NaH_2_PO_4_, 5 mM DTT, 0.25 M NaCl solution and proteinase inhibitor). The quantities of the extracted proteins were measured using the Lowry method. Five microliters each of protein standard (0, 2, 4, 6, 8, 10 μg/ml BSA) and samples was transferred into 96-well plates (triplicate), and 200 μl of solution A (2.5% SDS, 2.5% Na_2_CO_3_, 0.2 N NaOH, 0.025% CuSO_4_ and 0.05% tartaric acid) was added, and the plates were incubated for 30 min at room temperature. Then, 50 μl of solution B (20% Folin-Ciocalteu phenol reagent) was added, and the plates were incubated for 30 min at room temperature. Finally, the protein concentrations were measured at OD750 and calculated by comparison with the standards.

### SDS-PAGE and in-gel digestion

Fifty micrograms of protein was mixed with loading dye (77 mg/ml DTT, 0.1 g/ml SDS, 0.1 mg/ml bromophenol blue, 70% glycerol, and 0.4 M Tris, pH 6.8). Protein samples were separated by SDS-PAGE and stained with Coomassie blue. The gel was sliced into 15–18 pieces, and each gel plug was further cut into 1-mm^3^ cubes and transferred into low binding 96-well plates. Tryptic digestion was performed by incubating the gel pieces with 25 mM NH_4_HCO_3_ for 10 min, adding 200 μl of acetonitrile (ACN) and incubating for 10 min with shaking. After ACN removal, 50 μl of 10 mM DTT in 10 mM NH_4_HCO_3_ was added, and the gel pieces were incubated at 56°C for 1 hr. Next, 50 μl of 100 mM iodoacetamide in 10 mM NH_4_HCO_3_ was added, and then, the gel pieces were incubated at room temperature for 1 hr in the dark. Then, all liquid was removed. Next, 2 cycles of adding 200 μl of ACN to the gel pieces and shaking for 5 min were performed, followed by the removal of all liquid. Enzymatic digestion was performed by adding 10 μl of enzyme solution (10 ng/μl trypsin in 10 mM NH_4_HCO_3_) to the gel pieces and incubating at 37°C for 3 hr. The peptide was extracted by 3 cycles of adding 50 μl of 50% ACN to the gel pieces and shaking at room temperature for 10 min. Finally, the peptide solutions were transferred into new low binding 96-well plates and dried at 40°C. The dry peptides were kept at -20°C until analysis.

### LC MS/MS analysis

The peptide samples were resuspended in 0.1% formic acid, mixed with a pipette 100 times and transferred into low-binding tubes. The samples were centrifuged at 8,000×*g* for 10 min and transferred into vial tubes. Then, 4.5 μl of peptide sample was injected into a LC MS/MS analyzer (SYNAPT HDMS mass spectrometer, Waters, UK). Nanoscale LC separation of tryptic peptides was performed using a nanoACQUITY system equipped with a Symmetry C18 5 μm, 180 μm x 20 mm Trap column and a BEH130 C18 1.7 μm, 100 μm x 100 mm analytical reversed phase column. The samples were initially transferred with mobile phase A solution (0.1% formic acid) added to the trap column at a flow rate of 15 μl/min for 1 min. The peptides were separated with a gradient of 15–50% mobile phase B solution (0.1% formic acid in acetonitrile) for 15 min at a flow rate of 600 nl/min, followed by a 3-min rinse with 80% mobile phase B. The column temperature was maintained at 35°C. The lock mass was delivered from the auxiliary pump of the nanoACQUITY pump with a constant flow rate of 500 nl/min at a concentration of 200 fmol/μl fibrinopeptide B to the reference sprayer of the NanoLockSpray source of the machine. For all measurements, the mass spectrometer was operated in the V-mode of analysis with a resolution of at least 10,000 full-width half-maximum. All analyses were performed using the positive nanoelectrospray ion mode. The time-of-flight analyzer of the mass spectrometer was externally calibrated with fibrinopeptide B from 50 to 1600 m/z with acquisition lock mass corrected using the monoisotopic mass of the doubly charged precursor of [Glu1], a fibrinopeptide B. The reference sprayer was sampled at a frequency of 20 sec. The trap energy was set at a collision energy of 6 V. In the transfer collision energy control, low energy was set at 4 V. The quadrupole mass analyzer was adjusted such that ions from m/z 300 to 1800 were efficiently transmitted. The MS/MS survey was over the range 50 to 1,990 Da and 0.5 sec scan time. The values were normalized using a BSA external intensity control.

### Western blot analysis

Protein samples (75 μg) were analyzed by 12.5% SDS-PAGE. The proteins were transferred onto nitrocellulose membranes (0.2 μm, Bio-Rad, USA) using blotting buffer (7.25 mg/ml Tris base, 3.6 mg/ml glycine, and 0.5 mg/ml SDS) and a blotting machine (Trans-Blot SD semi-dry transfer cell, Bio-Rad, USA) at 18 V for 30 min. The protein-containing membranes were washed twice for 5 min with PBS-Tween solution. The blotted membranes were blocked using 5% skim milk in a PBS-Tween solution at 4°C overnight. The specific proteins were detected by adding a primary antibody solution (rabbit anti-CAECAM at 1:500 dilution (Abcam, UK) and mouse anti-β-actin at 1:3,000 dilution (Sigma, USA)) to the blotted membranes, incubating for 10 hr and washing three times for 5 min each with a PBS-Tween solution. The secondary antibody solution (HRP-conjugated anti-β-actin (Abcam, UK) and anti-rabbit IgG for anti-CAECAM (Sigma, USA) at 1:1,000 dilution) was added to the blots, incubated for 2 hr and washed three times for 5 min each with a PBS-Tween solution. Enhanced chemiluminescence was employed using chemiluminescent reagents (Thermo Scientific, USA). The blots were detected and analyzed by Image Quant LAS 4000 software (GE Healthcare Life Science, UK).

### Bioinformatics and data analyses

DeCyderMS 2.0 differential analysis software (DeCyderMS, GE Healthcare Life Science, UK) was used to analyze the data from LC MS/MS for protein identification and quantitation. The intensities of peptide signals were analyzed using the PepDetect module. The peptides were matched across different signal intensity maps among the tested conditions using the PepMatch module. The relative abundances of peptides were expressed as log_2_ intensities, with the mass tolerance set to 0.5 Da and the retention time tolerance set to 1.0 min. All log_2_ intensities of the sample were normalized to the ion intensity distribution of BSA. An average abundance ratio > 2-fold higher than BSA external intensity control was determined to be an identified and expressed protein with a significant standard t-test p-value < 0.05. The analyzed data from DeCyderMS were submitted for a database search using Mascot software (Matrix Science, London, UK). The data were searched against the NCBI database for protein identification. Database interrogation was as follows: taxonomy (human or eukaryote), enzyme (trypsin), variable modifications (carbamidomethyl, oxidation of methionine residues), mass values (monoisotopic), protein mass (unrestricted), peptide mass tolerance (± 1.2 Da), fragment mass tolerance (± 0.6 Da), peptide charge state (1+, 2+, and 3+), and max missed cleavages. Group-to-group comparisons were performed by linear regression. Statistical analyses were performed in R using the limma, gplots, VennDiagram and statmod packages. We used the lmFit and eBayes functions in limma to compare groups and calculate moderated t-statistics. The false discovery rate was controlled with the Benjamini-Hochberg method. The network analyses were performed using Ingenuity software (Qiagen, Germany) and computes a network score according to the fit of that network to the user-defined set of focus genes. The score is the −log10(p-value) of a Fishers-exact test and hence indicates the likelihood of the focus genes in a network are not being found together due to random chance.

## Results

**Fig 1:**
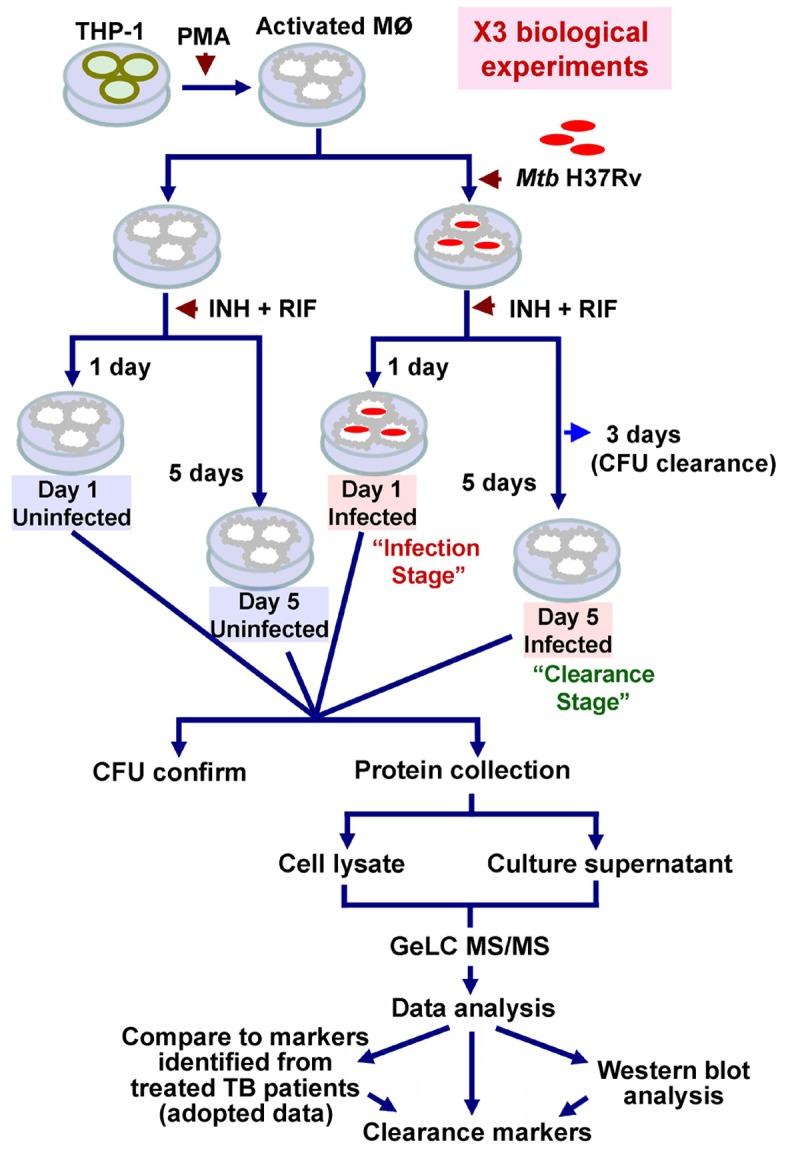
General work flow. THP-1 cells were activated using 50 nM PMA and infected with *Mtb* H37Rv. The infected macrophages were treated with 3 μg of INH and 9 μg of RIF for 1 day (Day 1 Infected) (*Mtb* remaining in the cells, *i*.*e*., infection stage) and 5 days (Day 5 Infected) (no *Mtb* remaining in the cells 2 days after clearance, *i*.*e*., clearance stage). *Mtb* clearance was confirmed by CFU determination at 3 days post-infection. THP-1 cells treated with drugs (without *Mtb*) for 1 day (Day 1 Uninfected) and 5 days (Day 5 Uninfected) post-infection were used as background controls. The culture supernatant and cell lysates were collected. CFU counts were performed to confirm the clearance stage of *Mtb* in intracellular and extracellular compartments from all experiments. Three biological replicates of the experiments were performed. The proteomes were analyzed by GeLC MS/MS. A western blot was performed to validate the proteins identified by GeLC MS/MS. The candidate clearance markers were compared to markers from patients treated with anti-TB therapy from previous studies.

To identify protein markers of *Mtb* infection or clearance, we systematically assessed the intracellular and secreted proteomes of PMA-activated THP-1 cells infected with *Mtb* H37Rv and treated with INH/RIF for one or five days compared to uninfected cells treated with INH/RIF (Fig 1). The correlation between independent biological replicates was moderate (mean *R* ^*2*^ value = 0.48), regardless of the infection condition and time (S1 Fig). The total numbers of unique peptides and named proteins identified from the LC MS/MS analysis were 4,440 and 3,291, respectively.

### Patterns of protein expression during treated infection before and after *Mtb* clearance identify potential markers of infection and clearance

**Fig 2:**
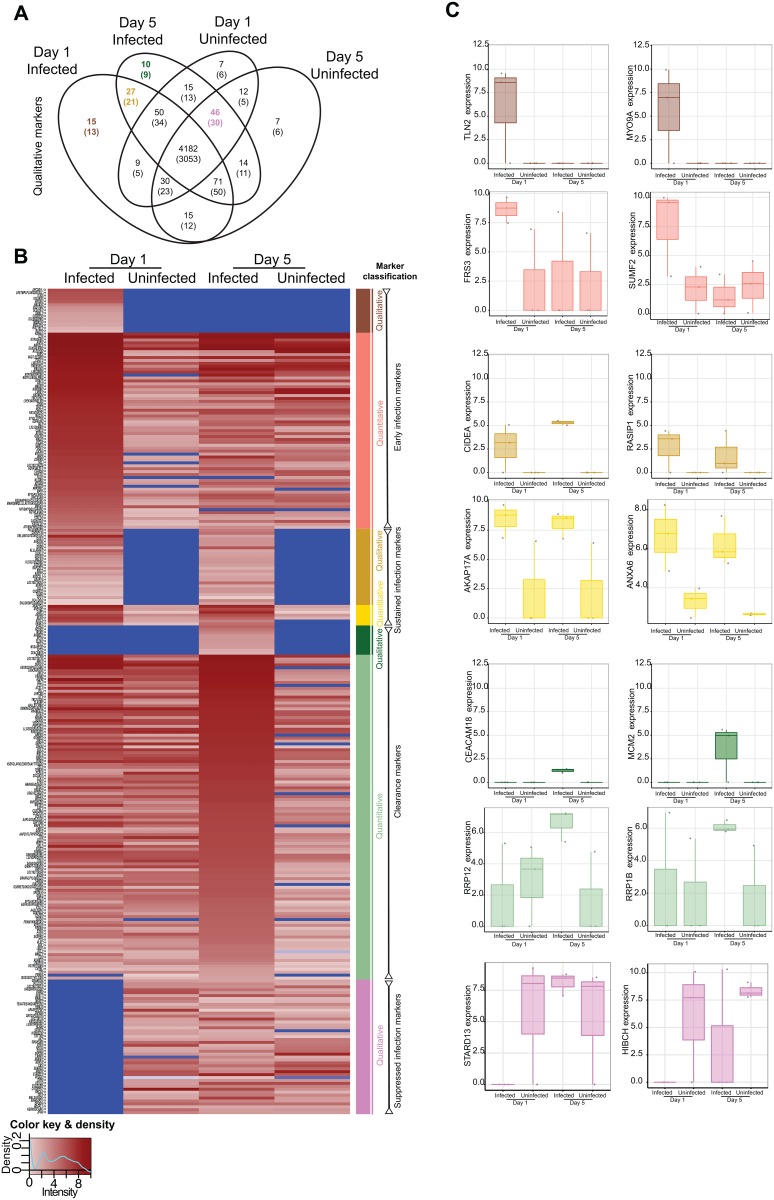
Proteome comparisons among conditions. (**Figure A**) Venn diagram of the proteome showing the number of unique peptides detected according to each condition. The number in brackets refers to the number of unique identifiable proteins to which the peptides match in the database. Consideration according to presence/absence defined the qualitative markers, see also Table 1. (**Figure B**) Heatmap depicting the level of expression (absent in blue, lowest in light pink, and highest in burgundy according to the key at the bottom left) across conditions as demarcated at the upper part of the heatmap. Protein names, or peptide names if no match was found for a peptide, are shown to the left, where the symbols ‘<>‘ or ‘><‘ denote detection in the secreted or intracellular proteome, respectively. Colored bars at the right of the heatmap correspond to the classification into seven classes according to qualitative or quantitative criteria. (**Figure C**) Examples of peptides from each class (denoted by color) of marker are shown with the expression level according to the condition. Each replicate is denoted by a point, and boxes show the median (central line), IQR (outer box) and range (whiskers) in the expression level. The y-axis refers to the protein to which the peptide maps.

After LC MS/MS analysis, 2,889 unique peptides from the intracellular proteome, 1,481 peptides from the secreted proteome and 70 peptides common to both the intracellular and secreted proteomes were identified. Considering the total proteome (intracellular and secreted combined), 91.3% (4,182/4,510) of peptides were common to all four treatment*time comparison groups (Fig 2A). We classified peptides qualitatively according to the presence or absence of expression during each condition and quantitatively according to significant differences in level of expression for proteins that were present in both comparison groups. The definitions used for classification are shown in Table 1. We note that the qualitative markers are wholly absent/present and so represent the most robust markers for further follow-up *in vivo*, whereas the quantitative markers are likely more useful in exploring the host-microbe interaction at play as they provide additional plausible molecules that may be involved. The quantitative markers should therefore be regarded as hypothesis generating in view of the multiple-hypotheses tested.

**Table 1:** Classification of peptides according to qualitative or quantitative differences between activated THP-1 cells infected with *M*. *tuberculosis* H37Rv or left uninfected and sampled at Day 1 or Day 5.

Classes	Qualitative definitions	Quantitative definitions
Early infection markers	Detectable in infected Day 1 cells only	Significantly more highly expressed at infected Day 1 vs uninfected Day 1 cells, but not significantly more highly expressed at infected Day 5 vs uninfected Day 5 cells
Sustained infection markers	Detectable in infected Day 1 and Day 5 cells but not in uninfected cells	Significantly more highly expressed at infected Day 1 vs uninfected Day 1 cells and significantly more highly expressed at infected Day 5 vs uninfected Day 5 cells
Clearance markers	Detectable in infected Day 5 cells (i.e. after clearance) only	Significantly more highly expressed at infected Day 5 vs uninfected Day 5, but not significantly more highly expressed at infected Day 1 vs uninfected Day1 cells
Suppressed infection marker	Suppressed in Day 1 infected cells but present in uninfected cells and restored in infected Day 5 (i.e. after clearance)	-

We identified 15 peptides (13 named proteins) as “qualitative early infection markers”, 27 peptides (21 named proteins) as “qualitative sustained infection markers” and 10 peptides (9 named proteins) as “qualitative clearance markers” (Fig 2A). In addition to these peptides whose qualitative presence/absence may identify infection/clearance states, we classified 67 peptides (42 named proteins), 7 peptides (6 named proteins), and 111 peptides (85 named proteins) as quantitative early infection, sustained infection or clearance markers, respectively, according to significant differences in quantitative expression after adjustment for multiple comparisons (Fig 2B and S2 Table). In addition, we identified 46 peptides (30 named proteins) as “suppressed infection markers” because these peptides were suppressed during infection (day 1) but expressed in uninfected cells (day 1 and 5) and because their expression was restored when the infection was cleared from cells by day 5 (Fig 2B and S2 Table). Examples of the patterns of peptides/proteins in each class are shown in Fig 2C. Interestingly several markers are observed in both intra- and extra-cellular compartments, adding strength to their association. The raw data of intracellular (cell lysates) and extracellular (culture supernatant) proteomes from all independent experiments, time points and conditions are available in the supplementary tables (S3 and S4 Tables, respectively).

### Network analyses of early infection, sustained infection and microbiological clearance markers provide insight into the biology of the host-pathogen interaction

**Fig 3:**
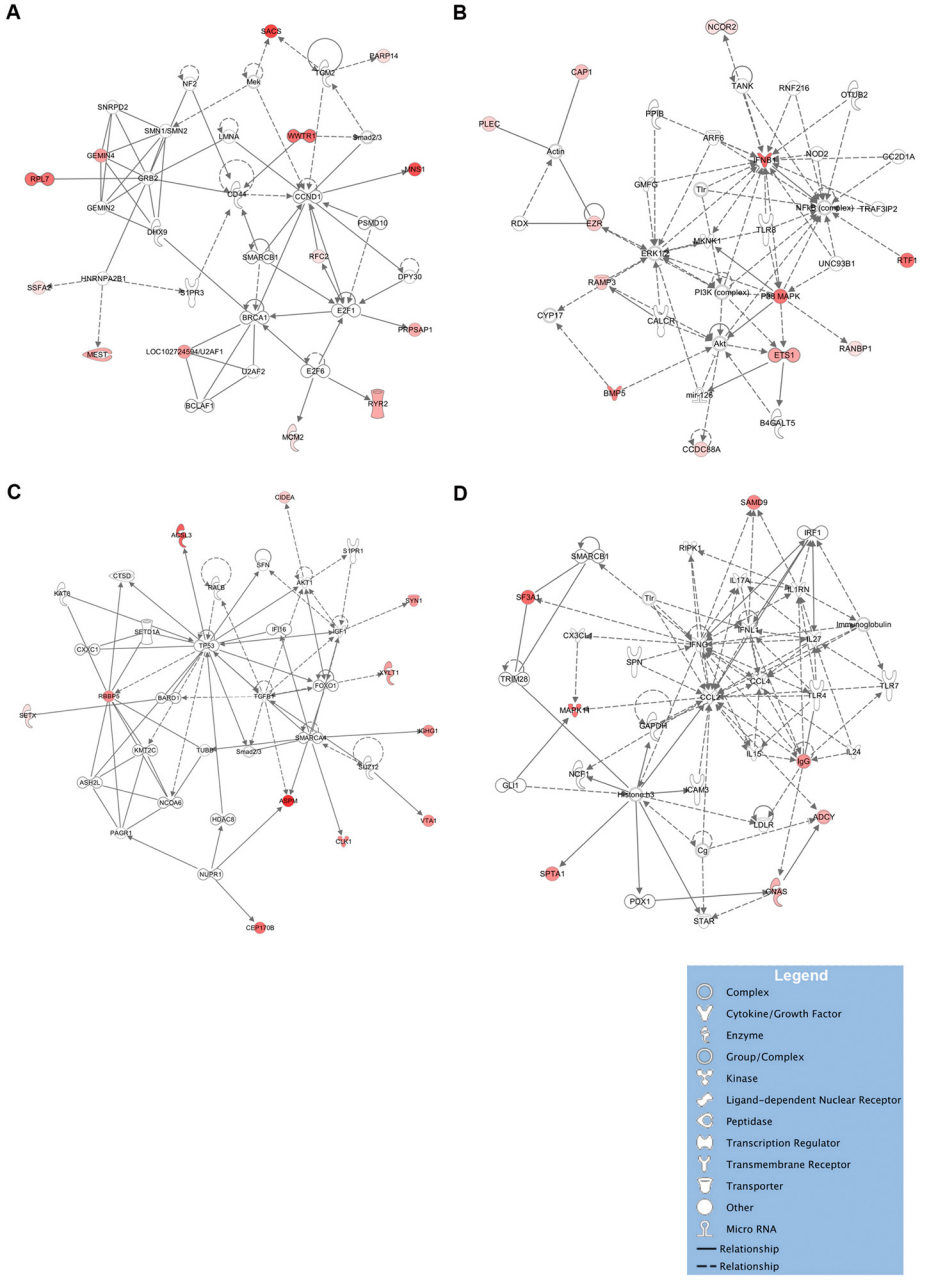
Network analyses of protein clearance markers. The majority of clearance markers belong to one of four networks. Network A centers on CD44 and CCND1 and consists of genes involved in the cell cycle and RNA post-transcriptional modification (**Figure A**). Network B centers on IFNβ1, NF-κB, ERK1 and MAPK and includes several additional genes involved in antimicrobial responses, such as TLR8 (**Figure B**). Network C centers on TP53, and TGF-β and is associated with the cell cycle and with proliferation (**Figure C**). Network D centers on CCL2, CCL4 and IFN-γ, which are associated with cell activation and migration and which play a central role in tuberculosis (**Figure D**). Solid lines denote a direct protein-protein interaction, such as binding; dotted lines denote other relationships, such as co-expression, regulation and activation, phosphorylation or cleavage relationships. The intensity of protein expression is denoted in shades of red proportionate to the level of expression.

A network analysis, leveraging protein-protein interaction data, of the clearance markers identified four networks of proteins that were significantly more likely than chance to be co-ordinately associated (Fishers exact test −log_10_p > 2) amongst clearance markers (Fig 3). (i) Thirteen proteins were identified in a network (−log_10_p = 21) centered on CD44 and CCND1, which is enriched for disease and functional annotations related to the cell cycle (p = 1.7x10^-6^) and with RNA post-transcriptional modification (p = 9x10^-7^) (Fig 3A); (ii) 12 proteins were in a network (−log_10_p = 21) centered on IFN-β1, NF-κB, and MAPK (Fig 3B), which is enriched for annotations related to with antimicrobial and inflammatory responses (p = 4.6x10^-6^); (iii) 11 proteins were in a network (−log_10_p = 17) centered on TP53 and TGF-β (Fig 3C), which is enriched for annotations related to DNA replication, recombination and repair (p = 1.3x10^-8^), cell-to-cell signaling and interaction (p = 1.5x10^-7^) and cell growth and proliferation (p = 1.5x10^-7^); and (iv) 7 proteins were in a network (−log_10_p = 6) centered on CCL2, CCL4 and IFN-γ (Fig 3D), which is enriched for annotation related to cell death and survival (p = 2x10^-14^), inflammatory response (p = 7.9x10^-13^), immune cell trafficking (p = 1.2x10^-12^). Moreover, these networks are linked through common proteins, such as TLR, smad2/3, and SMARCB1.

Among the early infection markers, two networks (−log_10_p = 31 and −log_10_p = 15) centered on MYC and MET respectively were significant (S2A and S2B Fig). For sustained infection markers, a network (−log_10_p = 21) converging on the NF-kB complex and TP53 was identified (S2C Fig). For suppressed infection markers, one network (−log_10_p = 9) converging on BRCA1 and BRCA2 was identified (S2D Fig).

### Validation of the presence of proteins by western blot analysis

**Fig 4:**
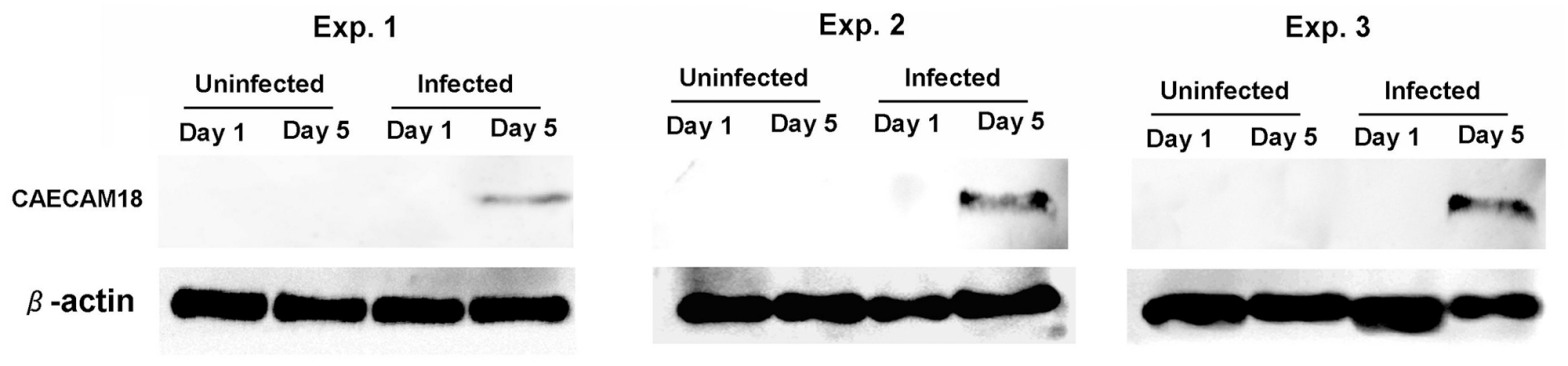
Validation of proteomic analysis by western blot using a monoclonal antibody to CAECAM 18. CAECAM was uniquely detected in all 3 independent experiments.

To validate the proteins identified by GeLC MS/MS analysis, CAECAM18, a putative clearance marker, was selected for western blot analysis. CAECAM18 was selected due to consistent results among replicates (narrowest SD) and due to the lowest intensity of all clearance markers. We reasoned that validating the weakest clearance marker may support the sensitivity of the MS/MS approach in the absence of the ability to perform western blots for all markers. β-Actin was used as a normalizing control. The band intensity of the tested proteins corresponded well to the LC MS/MS analysis results (Fig 4).

## Discussion

Biomarkers indicating *Mtb* infection clearance could facilitate treatment monitoring for both active TB and LTBI and identify individuals who successfully clear infection following exposure. Several previous studies have identified proteomic markers for LTBI and active TB [9, 10, 12, 18]. Studies exploring TB treatment outcome markers have used clinical evidence and microbiological evidence, particularly sputum microscopy and culture [19, 20]. Other studies have applied molecular and serological markers for treatment monitoring [21–24]. However, no studies have identified biomarkers of microbiologically-confirmed sterilized *Mtb* infection. We used an *in vitro* model of treated *Mtb* infection coupled with GeLC MS/MS to identify and quantify peptides that may indicate *Mtb* infection clearance. We compared the proteomes between infection and clearance stages and found protein signatures that were associated with early infection, sustained following infection, suppressed during infection or mycobacterial clearance. Our findings provide a rich, list of candidates for further clinical validation as *Mtb* clearance markers after anti-TB treatment. The identification of both qualitative and quantitative markers further provide prioritization for these potential biomarkers.

Clearance markers expressed only after mycobacterial clearance from host cells and not found during early infection of cells or in uninfected controls were associated with the cell cycle, RNA post-transcriptional modification, antimicrobial responses, proliferation, cell migration and movement. The networks for the cell cycle and for RNA post-transcriptional modification centered on CD44 and CCND1. CD44 is a macrophage binding receptor that mediates macrophage recruitment and protective immunity against TB [25]. CD44 knockout mice have enhanced susceptibility to *Mtb* infection due to a lower rate of neutrophil migration [26]. Another chronic infection, hepatitis C virus infection, interferes with CCND1 (Cyclin D1) expression and with the cell cycle; similar pathways may be involved in *Mtb* infection [27]. Another network centered on chemokines (CCL2 and CCL4) and IFN-γ also supports a role for cell recruitment and immunity in infection. After *Mtb* clearance, the immune-related cellular activity and recruitment of infection-experienced cells appears to be greatly activated. The network of proteins involved in antimicrobial and inflammatory response centered on IFN-β1, NF-κB and MAPK is consistent with IFN-β1’s role against *Mtb* infection in infected macrophages [28] and in rhesus macaques [29]. Virulent *Mtb* strains inhibit IFN-β1 expression [30]. Similarly, MAPK, which is involved in transducing TLR signals *via* matrix metalloproteinase (MMP) to recruit immune cells to an infected site [31], is also directly antagonized by a 19-kDa cell wall component of *Mtb* [32]. Several additional genes involved in antimicrobial responses, such as TLR8, which is known to be involved in TB disease [33], were also upregulated after clearance. The proteins from these networks were activated, indicating an increase in the anti-*Mtb* response in infection-experienced cells. Another network of cell cycle- and proliferation-related proteins centered on TP53 and TGF-β was enriched. TP53 and TGF-β have functions in cytostasis, apoptosis and autophagy signaling pathways [34, 35]. The activation of this network after clearance may indicate a homeostatic mechanism in infection-experienced cells and it is concordant with the regulatory function of IFN-β1 in inhibiting the IFN- γ pathway through the induction of IL-10 [36]. Taken together, infection-experienced cells appear to adapt cellular processes to increase anti-*Mtb* responses, immune cell recruitment and homeostatic mechanism after infection.

The expression pattern of clearance markers is consistent with some of these markers being involved in trained innate immunity [37]. Notwithstanding the obvious differences between our reductionist model here and *in vivo* models which may be potentially confounded by the different cell types, tissue compartments, patient comorbidities, we compared the candidate clearance markers described here to transcriptional markers from patients treated for 26 weeks treatment with anti-TB drug [38] and serum protein markers from patients treated with anti-TB therapy for eight weeks [16]. Notably, three of the clearance markers in our study, SSFA2, PARP14, PSME4, overlap with transcriptional markers identified from TB patients after 26 weeks of treatment with anti-TB drugs [38]. Treatment with anti-TB drugs for 26 weeks may better resemble our experiment in which *Mtb* was sterilized from cells, unlike eight week-treated hosts [16], in whom *Mtb* may remain. In our study, SSFA2 (sperm-specific antigen 2) was detected after mycobacterial clearance but was undetectable in infected or uninfected samples. Moreover, SSFA2 showed the largest fold change in expression. SSFA2 was associated with caloric restriction in a rhesus macaque study [39]. However, its function, particularly in immunity and infection, is unknown and requires further exploration.

The majority of the identified early infection markers belong to two networks of molecules involved in antimicrobial response and apoptosis. The first network centers on hepcidin antimicrobial peptide (HAMP) and Myc. Myc expression was shown to be associated with the induction of TNF-α and IL-6 and suppression of intracellular mycobacterial growth [40]. Hepcidin may function as an antimicrobial peptide against *Mtb* [41, 42]. Another notable network centered on CASP3 and Akt is linked to apoptosis regulation. CASP3 has been shown to be involved in inducing apoptosis during *Mtb* infection [43], whereas Akt reduces apoptosis and enhances *Mtb* survival [44]. The counteracting proteins associated with apoptosis may indicate the competitive polarity of the host-*Mtb* interaction. Taken together, the proteins associated with early infection portray the host’s response to *Mtb* infection by inducing antimicrobial response and apoptosis. Alternatively, *Mtb* induces host immune suppressors such as the anti-apoptotic factor Akt to increase its intracellular survival. A network analysis of proteins that continue to be expressed after infection (sustained infection markers) was observed and centered on NF-κB and TP53, both of which are also counteracting factors for apoptosis [45]. Therefore, the counteracting proteins for apoptosis, i.e., CASP3 *vs*. Akt and NF-κB *vs*. TP53, both reflect the host-*Mtb* interaction in our study.

Additionally, we identified suppressed infection markers, *i*.*e*., the markers that were suppressed during infection and restored after *Mtb* was cleared from cells. In contrast to the clearance markers, the suppressed infection markers were restored after the cells experienced infection. The network analysis of suppressed infection markers centered on BRCA1 and BRCA2, which have roles in cell cycle control and DNA repair [46]. One possible interpretation is that *Mtb* infection suppressed these proteins to inhibit cell proliferation for its survival.

Recently, a clearance model of *Mtb* was suggested [37]. After alveolar macrophages are exposed to *Mtb*, they can eliminate *Mtb* by phagocytosis and/or recruit other innate cells for *Mtb* clearance, called “early clearance”. The inability to clear *Mtb* by innate immune mechanisms may lead to granuloma formation and adaptive host immune responses for clearance, called “delayed clearance”. In addition to immune clearance, *Mtb* can be eliminated from host tissue by anti-TB drugs. In this study, we used activated THP-1 cells infected with *Mtb* and treated with INH and RIF at the minimum serum concentration used in TB treatment. Unlike pre- *vs*. post- treatment comparisons *in vivo*, our experiment used the drugs in all comparative conditions, avoiding confounding drug effects on the proteome. Our model also focused on the likely first cellular events in early clearance in the infected macrophages. Therefore, although our study is limited by the use of an activated THP-1 cells model as opposed to a systemic host model, this study allows the direct measurement of target cell-derived proteins. The simple cell line experiment is advantageous because of the possibility to maintain a standard substrate for three independent experiments in order to overcome constraints in reproducibility of GeLC MS/MS. The THP-1 cell in our experiment was stimulated by PMA treatment followed by two days resting in culture without PMA similar to the protocol suggested previously [47] which was shown to enhance the similarity to primary monocyte derived macrophages (MDM). The single cell type model also excludes interfering factors from other sources, for example, other tissues, as may be the case in human serum studies. Validation through the evaluation of treated TB patients is hampered by difficulties in confidently assessing *Mtb* clearance from tissues. Hence, animal studies may be promising models for *Mtb* clearance because *Mtb* sterilization can be ensured from all organ tissues. However, *Mtb* is primarily a human pathogen, and host-pathogen coevolution may result in discrepancies between animal and human models. Future experiments may be adjusted to reflect the actual host environments, such as multicellular cell culture. Additionally, the use of multiple strains covering lineages of *Mtb* could overcome the limitation of the use only H37Rv strain in our study. To reflect natural clearance, host immune factors such as cytokines or antimicrobial peptides could be used in the model instead of anti-TB drugs.

Although our clearance marker was not validated in the heterogeneous TB patients, a comparison with the markers found in the blood of 26 week-treated TB patients from a previous study [37] showed some overlapping markers, thus supporting our results. Highly expressed and potential clearance makers, such as SSFA2, can be further validated in a TB patient population during treatment. The validation of blood proteins as clearance markers is hampered by interfering substances from serum proteins and by many other cell types that may be less associated with lung biology. However, blood samples are a practical for determining *Mtb* clearance because they are normally collected to test alongside other general tests. Sputum and bronchoalveolar lavage, which are more reflective of the lung response to *Mtb* and hence clearance, can also be used to validate the clearance markers in further studies. We envisage that the use of clearance markers in combination or patterns may increase their sensitivity in clinically heterogeneous samples. Alternatively, the combination of suppressed infection markers and clearance markers may increase the ability to assess clearance.

Differentiation between people who have latent *Mtb* versus people were previously infected with *Mtb* (cleared case) could contribute to better TB and LTBI treatment. Such a tool or marker could potentially make mass treatment of LTBI in endemic settings feasible by narrowing the target group of LTBI patients who require treatment. Such markers may also help assess anti-TB drug and vaccine development.

In summary, several mechanisms of interaction between activated THP-1 cells and *Mtb* were identified. After cells experience infection, activated proteins associated with several mechanisms against *Mtb* infection, cell development and recruitment were found. The competitive polarity of apoptosis between host and *Mtb* was shown. We identified several proteins that could potentially be used as biomarkers for TB treatment monitoring.

## Supporting Information

S1 Fig Biological replicates show moderate correlation in LC MS/MS detection intensity regardless of time points and treatment conditions. The values at the upper left of each panel show R^2^ for each pair denoted in the key to the right of the figure. A mean R^2^ value of 0.48 was found among replicates regardless of the treatment conditions.
(TIF)

S2 Fig Network analyses of early- (A-B), sustained- (C), and suppressed- infection markers (D). Network A is associated with the antimicrobial response and includes genes such as HAMP and MYC (**Figure A**). Network B centers on CASP3 *vs*. Akt (**Figure B**) and Network C centers on NF-κB *vs*. TP53 (**Figure C**), which are both involved in and counteract each other in the apoptosis pathway. Network D centers on BRCA1 and BRCA2 (**Figure D**). The intensity of protein expression is denoted in shades of red proportionate to the level of expression.
(TIF)

S1 Table Optimization of drug concentrations for macrophage infection experiments. (DOC)

S2 Table Peptides identified according to the conditions and classification. This table contains the list of proteins corresponding to Fig 2B.
(XLS)

S3 Table Raw data of intracellular (cell lysates) proteomes from all independent experiments, time points and conditions. (XLS)

S4 Table Raw data of extracellular (culture supernatants) proteomes from all independent experiments, time points and conditions. (XLS)
