# Supplementary figures and images for: Comparative Proteomics of Activated THP-1 Cells Infected with Mycobacterium tuberculosis Identifies Putative Clearance Biomarkers for Tuberculosis Treatment
Source: PLoS One. 2015 Jul 27;10(7):e0134168. doi: 10.1371/journal.pone.0134168 (PMC4516286; doi:10.1371/journal.pone.0134168)

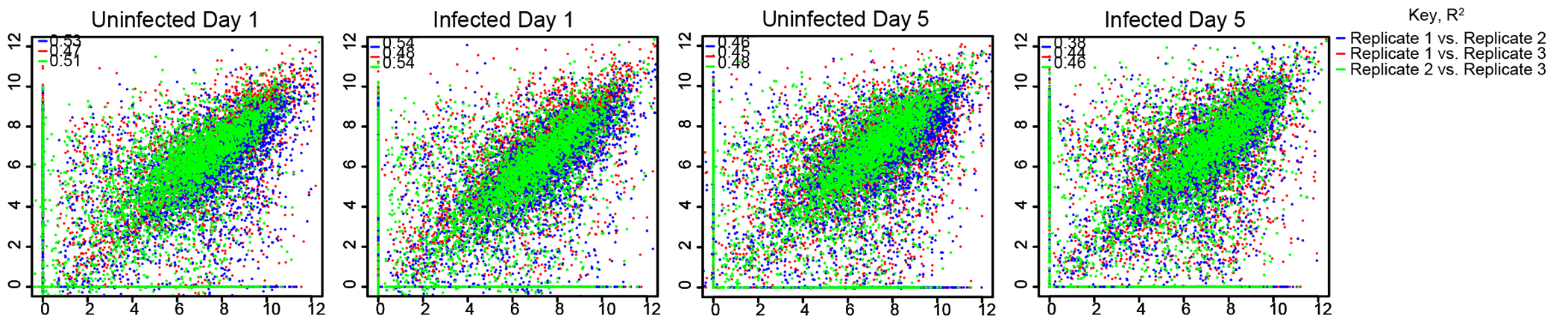

Supplement: S1 Fig — The values at the upper left of each panel show R2 for each pair denoted in the key to the right of the figure. A mean R2 value of 0.48 was found among replicates regardless of the treatment conditions. (TIF) [file pone.0134168.s001.tif]

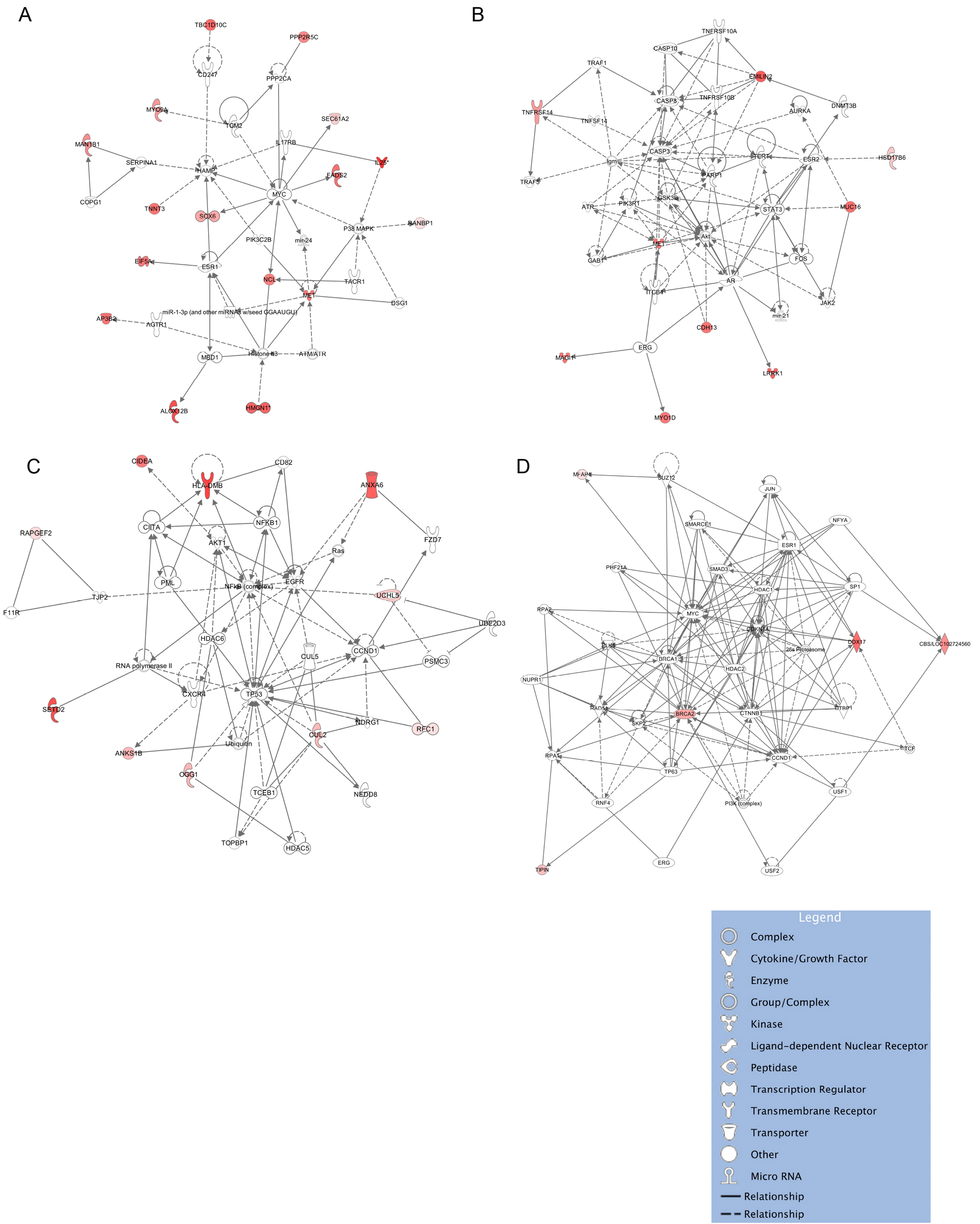

Supplement: S2 Fig — Network A is associated with the antimicrobial response and includes genes such as HAMP and MYC (Figure A). Network B centers on CASP3 vs. Akt (Figure B) and Network C centers on NF-κB vs. TP53 (Figure C), which are both involved in and counteract each other in the apoptosis pathway. Network D centers on BRCA1 and BRCA2 (Figure D). The intensity of protein expression is denoted in shades of red proportionate to the level of expression. (TIF) [file pone.0134168.s002.tif]
