# Supplementary material for: Comparative Proteomics of Activated THP-1 Cells Infected with Mycobacterium tuberculosis Identifies Putative Clearance Biomarkers for Tuberculosis Treatment
Source: PLoS One. 2015 Jul 27;10(7):e0134168. doi: 10.1371/journal.pone.0134168 (PMC4516286; doi:10.1371/journal.pone.0134168)
Supplement: S1 Table — (DOC) [file pone.0134168.s003.doc]

**S1 Table. Optimization of drug concentrations for macrophage infection experiments.**

| **Drug conc. (μg/ml)**  **INH:RIF** | **Day 1** | | **Day 3** | | **Day 5** | |
| --- | --- | --- | --- | --- | --- | --- |
|  | Intra | Extra | Intra | Extra | Intra | Extra |
| 0.25:0.75 | G | G | G | NG | NG | NG |
| 0.5:1.5 | G | G | G | NG | NG | NG |
| 1.0:3.0 | G | G | G | NG | NG | NG |
| 3.0:9.0* | G | NG | NG | NG | NG | NG |
| PC | G | G | G | G | G | G |
| NC | NG | NG | NG | NG | NG | NG |

Culture of THP-1 cells infected with *M. tuberculosis* H37Rv at MOI = 1 demonstrates intra- and extra-cellular microbial clearance based on CFU determination by day 3 post-infection when cells were treated with physiological concentrations of INH and RIF. PC = positive control for infection without drugs; NC = negative control for no infection; G = growth of *Mtb* that can be visually detected on M7H10 agar plate; Intra = intracellular; Extra = extracellular; NG = no growth of *Mtb* after incubation for 6 weeks*.* * Minimum concentration in the blood of tuberculosis patients.
